# Synthetic Biology Enables Programmable Cell‐Based Biosensors

**DOI:** 10.1002/cphc.201900739

**Published:** 2019-10-25

**Authors:** Maggie Hicks, Till T. Bachmann, Baojun Wang

**Affiliations:** ^1^ School of Biological Sciences University of Edinburgh Edinburgh UK; ^2^ Centre for Synthetic and Systems Biology University of Edinburgh Edinburgh UK; ^3^ Infection Medicine Edinburgh Medical School: Biomedical Sciences University of Edinburgh Edinburgh UK

**Keywords:** cell-based biosensor, genetic circuits, rational approaches, response curve, synthetic biology

## Abstract

Cell‐based biosensors offer cheap, portable and simple methods of detecting molecules of interest but have yet to be truly adopted commercially. Issues with their performance and specificity initially slowed the development of cell‐based biosensors. With the development of rational approaches to tune response curves, the performance of biosensors has rapidly improved and there are now many biosensors capable of sensing with the required performance. This has stimulated an increased interest in biosensors and their commercial potential. However the reliability, long term stability and biosecurity of these sensors are still barriers to commercial application and public acceptance. Research into overcoming these issues remains active. Here we present the state‐of‐the‐art tools offered by synthetic biology to allow construction of cell‐based biosensors with customisable performance to meet the real world requirements in terms of sensitivity and dynamic range and discuss the research progress to overcome the challenges in terms of the sensor stability and biosecurity fears.

## Graphical abstract

**Sensor in a cell**: This review presents the state‐of‐the‐art tools offered by synthetic biology to allow construction of cell‐based biosensors with customisable performance to meet the real‐world requirements in terms of sensitivity, selectivity, dynamic range and biosecurity.

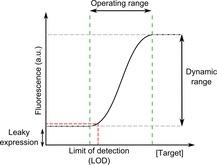

## 1 Introduction

Cell‐based biosensors harness a cell's natural ability to sense and respond to the environment by repurposing its sensing mechanisms in new genetic contexts, creating cells capable of detecting and producing a response to a specific molecule of interest. Cell‐based biosensors gained interest as an alternative method of sensing because they have several advantages over traditional methods including cost, portability, and the lack of equipment and trained personnel required for sensing. The flexibility of cell‐based biosensors in terms of the design and outputs available is another attractive feature because it allows biosensors to be tailored to the specific requirements for an application and preferred readouts. Cell‐based biosensors have potential in multiple areas of research, including environmental monitoring,^1^, ^2^ bioproduction,^3^, ^4^ biomedical applications in diagnostics^5^, ^6^ and health monitoring.^7^, ^8^

Despite the advantages, the development of successful commercial cell‐based biosensors has been slow due to several challenges hindering their construction and their ability to sense targets of interest at the relevant concentrations. For early cell‐based biosensors, optimisation of the initial constructs to improve the dynamic range and sensitivity was slow as the process was carried out ad hoc. The limited number of parts available also hindered development as many desired targets did not have known parts for sensing. Despite these challenges some sensors with the required performance were developed.^9^ The development of rational methods to tune biosensor performance and the increased number of available parts led to renewed interest in biosensors because the construction and optimisation has become much quicker. There now exists many examples of cell‐based biosensors which are able to detect disease markers, drugs, and environmental pollutants at their relevant concentrations.^1^, ^10^, ^11^ Despite the increasing number of biosensors in the literature capable of sensing relevant concentrations there are still very few commercial examples.^12^ This is because commercial cell‐based biosensors face challenges in acceptance arising from biosecurity fears, and concerns over the stability and reliability of the sensors and the methods for determining results.

This review aims to give an overview into current areas of potential applications, then examines the state‐of‐the art synthetic biology tools developed for improving the response of biosensors, the current research on expanding the range of biosensors and discusses the approaches currently being investigated to overcome the ongoing challenges of stability and biosecurity. The focus of this review is on prokaryotic cell‐based biosensors and the methods to tune their response. Other reviews and publications cover the methods of cell‐based biosensor design and response engineering for different approaches in more depth.^13^, ^14^, ^15^, ^16^, ^17^, ^18^

## 2 State‐of‐the‐Art of Cell‐Based Biosensor Applications

Cell‐based biosensors have been developed as potential alternative analytical devices for the detection of a wide range of molecules in various areas. Key areas have been bioproduction, medical and environmental monitoring due to the particular advantages biosensors offer in these areas.

Environmental monitoring has been a focus because biosensors can give information not only on the presence of pollutants but also on their bioavailability, which is important when considering the impact of the pollutant on the environment. Cell‐based biosensors also offer the possibility of remote testing for a pollutant which is a significant advantage when testing for dangerous materials such as explosive residue from mines.^11^

For medical applications cell‐based biosensors offer faster diagnostics than traditional methods, where culture of the infectious agent is commonly required as well as transport to a testing lab. More recently with the rise of interest in point‐of‐care testing and health monitoring wearable cell‐based biosensors have been developed to the proof‐of‐concept stage.^19^ The development of technologies such as microfluidics also mean that biosensors can be used in a high throughput manner which is highly important for identification of new drugs^20^ or drug resistance.^21^, ^22^ Cell‐based biosensors also allow the detection of a pathogen to be linked to downstream processes such as the production of a treatment.^23^

Bioproduction is a large area of research because it has the potential to allow the production of commercially important chemicals in a cheap and environmentally friendly way. However the yield from bioproduction methods can be highly variable depending on the cell culture health and metabolite availability. Recent work has focussed on the development of biosensors for metabolite availability^24^ and the physiological status of the cells,^3^, ^4^ to improve yields and make yields more similar between different batches.

Food safety applications have also been studied but less so, with the focus here on the detection of a molecule of interest which must not to be present in food commonly allergens and pathogens.

More recently using cell‐based biosensors to create functional materials has become an area of focus. These would allow materials to not only gain functionality but potentially regenerative abilities.^25^ Table 1 highlights the range of areas for which biosensors have been developed, giving recent examples.

**Table 1:** Representative applications of cell‐based biosensors. The table summarises some of the recent examples of synthetic biology enabled cell‐based biosensors in the literature over different areas of monitoring.

Application	Examples	References
Environmental monitoring	Detection of explosive residue	11
	Heavy metals	1, 26, 27, 28
	Pesticides	29, 30
	Pharmaceuticals	31
	Endocrine disruptors	32
	Hydrocarbons	33, 34
Toxicity assay	Whole‐cell human toxicity sensor	35
Diagnostics	Quorum sensing molecules for infection	5
	Gut inflammation	7, 36
	Sepsis	6
	Antimicrobial resistance	21, 22
Point‐of‐care monitoring	Proof‐of‐concept wearable device	19
Treatment	Detecting and supressing cholera	23
	Drug screening	20
Nutrition	Zinc monitoring in human serum	8
Bioproduction	Lactate production in cell culture	3
	Cell‐based stress	4
	Identification of optimal bioprocess parameters	24
Food safety	Pathogens	37
	Allergens	38
Functional biomaterials	Proof‐of‐concept biomaterial to detect chemical inducers such as IPTG and DAPG	25

## 3 Engineering Biosensing Parts

**Figure 1:**
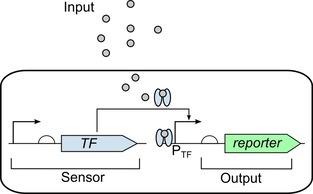
Biosensor architecture: A cell‐based biosensor works by the input entering the cell being detected by the sensor component (sensor) of the genetic circuit which is a gene constitutively expressing a transcription factor (TF) which then activates the generation of a reporter protein (output) by binding and activating the promoter responsive to the transcription factor (P_TF_).

Cell‐based biosensors follow the same general architecture, outlined in Figure 1, of a sensor encoded within a cell to detect the input which can then activate the output. This generic architecture has been employed in a wide range of different areas of sensing using many different outputs, although optical detection is the main focus for current biosensors. Synthetic biology has allowed the rapid construction of whole‐cell biosensors which can be programmed to produce the desired response.

To be able to construct a biosensor for a target of interest, parts which can be used to sense and respond to the target of interest first need to be identified. Initially, identifying parts which could be used was mostly due to chance. Part mining has been an important tool for identifying new parts for sensing molecules of interest. The increasing ease of sequencing has meant a large increase in the number of bacterial genomes available. Bioinformatics tools have been used to search these genomes for orthologues of known sensing elements by looking for closely related sequences. Parts have been identified both through searches based on DNA sequence similarity^39^ and by searching through part labels on protein databases.^40^

Directed evolution has also been used for generating new parts to build biosensors. Mutagenesis is used to create a library of mutants which are screened for the desired characteristics. This process is repeated until the optimal characteristics possible are obtained. Directed evolution has been successful in the generation of new or improved protein parts.^41^, ^42^ In general synthetic biology approaches to biosensor construction has not favoured the use of directed evolution to generate or improve parts. The generation of rational approaches which are widely applicable to many different constructs is preferred.

**Figure 2:**
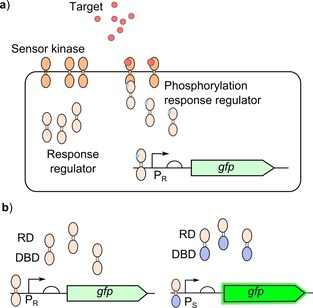
Bacterial two‐component systems for biosensing: a) The sensor module of the TCS sensor kinase binds to the target resulting in activation of the kinase domain, the response regulator is then phosphorylated by the activated kinase domain to activate the response regulator. The response regulator will then bind to the output promoter (P_R_) to generate the response. b) Response regulators containing their cognate receiver domain (RD) and DNA binding domain (DBD) will bind to their cognate output promoter (P_R_), response regulators with swapped DBD will then bind to an alternative output promoter (P_S_) which generates a stronger output.^47^

Two‐component systems (TCS) are a huge class of proteins responsible for sensing and responding to signals in nature.^43^ However they have not been frequently used when developing biosensors.^36^ This is due to difficulty in maintaining the activity of the components when moved into alternative bacterial species. The output promoters frequently have multiple methods of regulation, impacting the response. TCS contain a sensor module which binds to the target activating the kinase domain. The kinase domain then phosphorylates the response regulator to activate it resulting in binding to the output promoter to activate expression. The mechanism is outlined in Figure 2. Work into developing TCS for use in other contexts and species has looked at swapping sensor elements and has been carried out to improve TCS for use in biosensors. Work has focussed on altering the sensing domain of the sensor kinase so that the sensor kinase is able to detect different target inputs.^23^, ^44^, ^45^, ^46^ But the success has been limited due to differences in the regulation and interactions between different families. The response regulators of TCS are far more modular with much higher conservation in structure and regulation within TCS families, suggesting the DNA binding domains could be swapped between different TCS to allow different output promoters to be used, Figure 2b.^47^ This would allow the use of output promoters that are already well characterised and well understood to be used to control the expression of a reporter. Being able to easily repurpose TCS into biosensors will greatly expand the range of targets for which parts are already available.

**Figure 3:**
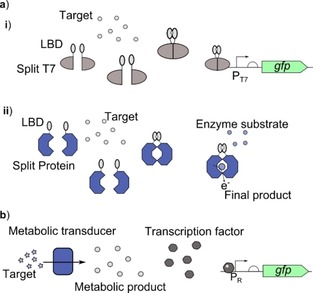
Approaches for universal cell‐based biosensor platforms: Universal biosensors aim to link specific recognition elements to a generic platform to expand the range of target molecules capable of being sensed through cell‐based biosensors, whilst generating a system that allows quick construction and optimisation of new cell‐based biosensors. a) Split protein biosensor systems work by adding the ligand binding domain (LBD) to each separate half of a protein which when brought back together generates a response. i) Split T7 polymerase (Split T7) is used to activate expression of a reporter protein from the T7 promoter (P_T7_).^48^ ii) Split glucose dehydrogenase is used to control the degradation of glucose releasing electrons which can be detected through a range of methods.^49^ b) The target molecule is broken down using a natural metabolic pathway (metabolic transducer) into a metabolic product which can be detected by a transcription factor to activate expression of a reporter from a responsive promoter (P_R_).^51^

Antibody derived parts such as nanobodies can be produced that bind to almost any target, so are a good method to bind to a target for which transcription factors and responsive promoters have not been found. These nanobodies can be combined with other parts to link the binding to the generation of a response. Split proteins, which when brought back together are capable of generating a response through activating transcription or an enzymatic reaction, can be brought back together through the binding of the target molecule by adding binding domains onto the split proteins.^48^, ^49^ This method has been used to try and generate universal cell‐based biosensor platforms to speed up development and optimisation by using a well understood system and is highlighted in Figure 3. A second method which uses antibody derived parts for detection has been developed where nanobodies can be expressed on the surface of bacterial cells which after binding to the target of interest result in agglutination.^50^ This method allows molecules which cannot enter the cell to be detected.

An alternative approach to split proteins is using metabolic by‐products as the molecule which is detected to generate the response. A metabolic pathway can then be used to generate this product from the real target of interest, so that this target can be sensed.^51^ The advantage of this method is quick optimisation as the response circuit chosen is already highly optimised. Both approaches to creating generic methods of cell‐based biosensing are shown in Figure 3.

Alternative sensing methods not using proteins have also been developed. DNA and RNA are capable of selectively binding molecules depending on 3D conformations which can be linked to outputs.^52^ DNA and RNA switches can also be used for detection to switch on transcription or translation.^53^

## 4 Tuning a Biosensor's Response

**Figure 4:**
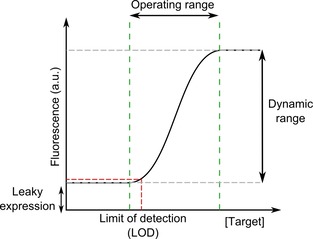
Characteristics of a typical biosensor response curve: The dynamic range is ratio between the minimum and maximum output expression (max/min ). The limit of detection (LOD) is the lowest concentration of the target which can be detected from the background response. The leaky expression is the level of reporter present when no target is present. These characteristics are commonly used to define the response of the biosensor. The operating range gives the concentrations of target which can be detected through a change in the output.

Early cell‐based biosensors showed poor response to target molecules as they frequently had highly leaky expression with a small dynamic range and poor sensitivity. These aspects of the response curve are highlighted in Figure 4. This was a problem because commonly targets of interest for sensing will be found in low concentrations and small changes in the concentration need to be detected. This requires high sensitivity and a good signal‐to‐noise ratio which means a good dynamic range is important. The specificity of early sensors was also an issue as many natural transcription factors show promiscuity. Biological samples are highly complex with many different molecules present that have the potential to affect the response.

Initially cell‐based biosensor optimisation was undertaken in an ad hoc manner, altering different parts of the genetic circuit and observing the effect. So optimisation methods were specific to each case. This did not improve approaches for developing new cell‐based biosensors with better characteristics as it required the process to be repeated for each new sensor. Synthetic biology has looked to develop rational methods capable of targeting a specific characteristic of the response curve. Mathematical modelling has been important in identifying the role specific parts within the sensor play in determining the response,^54^ so that by choosing certain parts the curve characteristics can be targeted individually.^55^ These methods have allowed more purposeful design and optimisation of cell‐based biosensors by following rules identified from other cell‐based biosensors’ behaviours. The methods identified for optimising the response of biosensors have focussed on protein based circuits, but the underlying reasoning also applies to DNA and RNA based sensors. The focus of the review is on bacterial cell‐based biosensors but the tools outlined for optimising the response can also be used to optimise eukaryotic cell‐based biosensors, and some examples are cited throughout the review.

### 4.1 Lowering the Limit of Detection

The limit of detection (LOD) is important for use in real life applications because molecules of interest are often present at very low concentrations therefore the lowest concentration that can be sensed needs to be able to go below the minimum concentration to be detected. This ensures that the relevant concentration range can be detected.

**Figure 5:**
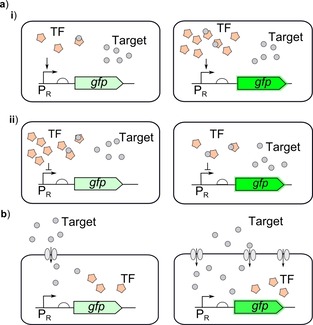
Tuning biosensor response through optimisation of intracellular receptor densities: a) By altering the intracellular concentration of the ligand‐responsive allosteric transcription factor (TF) the output from the response promoter (P_R_) can be altered. i) For activators, increasing the concentration of transcription factor can increase output from the response promoter making it more sensitive to the target molecule. ii) For repressors reducing the intracellular concentration of the transcription factor can increase the output from the response promoter making it more sensitive to the target molecule.^56^, ^57^ b) Altering the intracellular concentration of the target molecule can also be used to alter the response of the biosensor. The intracellular concentration can be increased by engineering import machinery to increase transport into the cell to increase the response.^26^

The intracellular concentration of the transcription factor regulating expression plays a large role in determining the concentration of the target which can be sensed. Altering the transcription factor concentration can be used to optimise the LOD, and depending on whether the sensor is an activator or repressor will determine whether the optimal response requires increasing or decreasing the intracellular concentration of the transcription factor,^56^, ^57^, ^58^ outlined in Figure 5a.

Improving the intracellular concentration of the target will improve sensitivity as this make more of the target available to the sensors. This can be achieved by adding or increasing import machinery outlined in Figure 5b,^26^ or by preventing the export of the target by deleting the cognate export machinery.

### 4.2 Modulating Dynamic Range

The dynamic range is the ratio between the leaky expression and the maximum expression of the reporter. Maximising the dynamic range is important for being able to reliably determine the result from a biosensor ensuring a good signal‐to‐noise ratio. To maximise the difference leaky expression needs to be minimised whilst increasing the maximum expression as much as possible.

**Figure 6:**
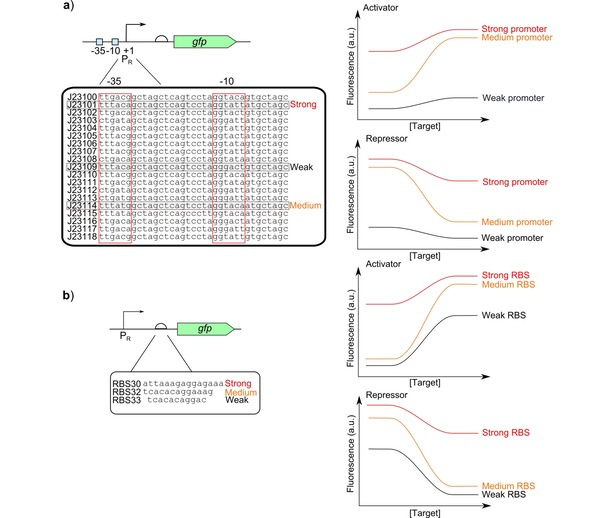
Part engineering to optimise dynamic range: a) Anderson collection of promoters (http://parts.igem.org/Promoters/Catalog/Anderson) are well characterised constitutive promoter sequences of a range of strengths which could be used to replace the −35 and −10 sites of responsive promoters to alter their behaviour. Different strength promoter sequences will alter the maximum expression and the leakiness. Response curves are shown for activator and repressor systems.^59^ b) Alternative ribosome binding site (RBS) sequences of different strengths can be replaced to alter the response of the biosensors.^60^, ^61^ The response curves for activator and repressor systems with different RBS sequences are shown.

The promoter responsible for controlling the expression of the output can be used for optimisation and improving the dynamic range by altering both the leaky expression and the maximum expression. Strong promoters will lead to high leaky expression without a large change in expression on activation, whilst a weak promoter will give low leaky expression but also have low maximum expression shown in Figure 6a.^58^, ^59^ This is due to the binding equilibrium between the polymerase and the promoter. Increasing the binding constant too much means the polymerase is able to bind well even without the transcription factor whilst the opposite is true for reducing the binding constant. The translation of the mRNA produced can also be optimised to improve the final output. A high translation rate will lead to higher leaky expression as well as increasing the maximum expression, whilst a low translation rate will reduce leaky expression but also the maximum expression shown in Figure 6b.^60^, ^61^ The simplest method of altering the translation rate is to change the RBS strength. Initially libraries were generated for screening these small parts and it was possible to explore all the variants. Now these parts can now be quickly optimised and tested as a result of the development of standard well characterised parts (http://parts.igem.org) and can be easily substituted, so increasing the speed of optimisation. Specific translation rates and the RBS sequences required to generate these rates can also now be predicted.^62^

**Figure 7:**
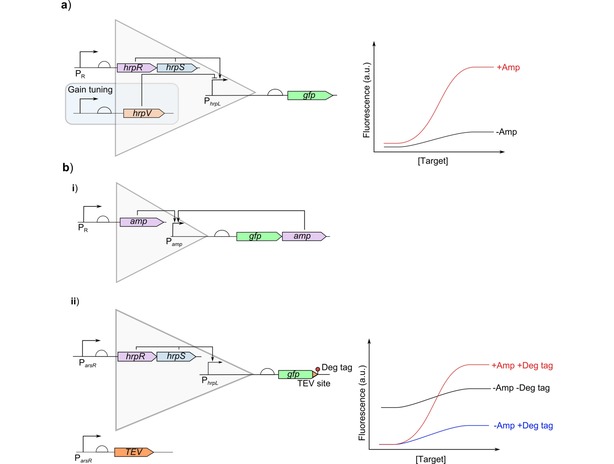
Biological signal amplification to boost dynamic range: Genetic amplifiers are ligand‐free ultrasensitive transcription factor and promoter pairs which have a large output dynamic range and can be linked to sensing transcription factors and promoters with low dynamic ranges to increase them. a) The simplest architecture of an amplifier is an activator system with high expression where the responsive promoter (P_R_) is used to control the expression of the activator (hrpS and hrpR) which then activates the amplifier promoter (P_hrpL_). The effect of an amplifier on the response curve is shown by the two curves. The level of amplification (amplification gain) can be tuned by adding another level of regulation to the amplifier through a repressor (hrpV).^63^ b) Alternative architectures can also be used for amplification. i) Positive feedback can be added to increase the amplification on induction placing the amplifier protein (amp) under the control of the amplifier promoter (P_amp_) and the responsive promoter (P_R_).^64^ ii) Amplifiers have been combined with other techniques to prevent increased leaky expression due to amplification.^1^ The addition of a degradation tag to the output protein (P_arsR_) reduces basal expression. Whilst the expression of a protease under the control of the same input promoter can cleave off the degradation tag when the target is present to prevent reduction in the maximal expression.

Genetic amplifiers can be used to boost the response of a cell‐based biosensor by using ligand‐free transcription factors which generate strong expression from their cognate promoter under the control of the promoter responsive to the target. The amplification methods are shown in Figure 7. The simplest genetic amplifiers use transcriptional activators which can then be tuned to control the amplification gain using additional regulators to the amplifier's promoter, Figure 7a.^1^, ^28^, ^63^ Positive feedback has also been used to increase the gain when activated, Figure 7bi.^64^ This signal amplification also improves the LOD by maximising the change in response between different concentrations. Genetic amplifiers are a good generic method to increase expression. However as any basal expression will also be amplified, amplification tends to be used after other optimisation methods. First the leakiness need to be minimised as much as possible to prevent basal amplification being an issue. Combinations of genetic amplifiers with methods for reducing leakiness have been used to maximise the dynamic range by amplifying the maximal expression whilst preventing amplification of basal expression Figure 7b**ii**.^1^

### 4.3 Managing Leakiness

High leaky expression is frequently an issue when developing cell‐based biosensors. There are several methods for reducing leaky expression which act on transcription and translation of the reporter, such as the use of degradation tags, antisense transcription and altering the operator sites.

**Figure 8:**
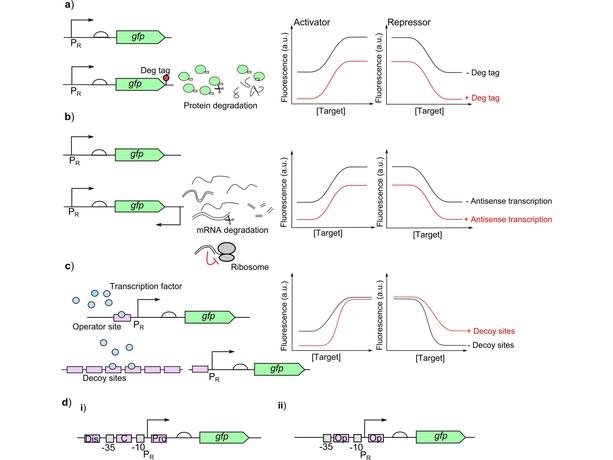
Approaches to reduce leaky expression: a) Degradation tags (Deg tags) can be used for post‐translational reduction of leaky expression. Degradation tags increase the degradation of the reporter protein and reduce the levels within the cells. The strength of the degradation tag will determine the reduction in the levels of the reporter protein.^8^, ^61^ The two curves show the change in the response curve with a degradation tag (+ Deg tag) or without (− Deg tag) for activators and repressors. b) A downstream promoter placed on the non‐coding strand can be used to produce a complementary RNA to the mRNA to generate a double stranded complex which is degraded or blocks the ribosome to prevent translation. The level of mRNA prevented from being used in translation is controlled by the level of complementary RNA which can be altered by changing the strength of the promoter used to express the complementary RNA.^66^ The two curves show the change in the response curve with antisense transcription (+ Antisense transcription) or without (− Antisense transcription) for activators and repressors. c) Decoy sites titrate the binding of the transcription factor away from the responsive promoter (P_R_). For activator systems this can reduce leakiness by preventing binding of the transcription factor in the absence of target.^8^, ^67^ Whilst for repressors this can be used to reduce the amount of effective repressor. The two curves show the change in the response curve with decoy sites (+ Decoy sites) or without (− Decoy sites) for activators and repressors. d) For repressors the position and number of operator sites can be altered to improve the efficiency of the repressor to reduce leaky expression. i) There are three possible sites for the where the operator can be. First the core site (C) is most efficient and its efficiency is increased when the binding site overlaps with either the −35 or −10 regions.^59^ Second is the proximal site (Pro) downstream of the transcription start site where the repressor can act as a physical block to the polymerase followed by the distal site (Dis).^68^ ii) If the initial operator site (Op) cannot provide enough repression then an additional site can be added downstream to act as a physical ‘roadblock’.^69^

Degradation tags on the output protein is a simple method for reducing leakiness post‐translationally, shown in Figure 8a.^8^ These increase the turnover of the reporter protein so without activation of expression the leaky level is kept to a minimum, but this will also affect the maximum. Changing the strength of the degradation tag can help optimise this effect. Adding a cleavage site between the degradation tag and the protein whilst controlling the expression of the protease using the responsive promoter can be used so that degradation is prevented when expression from the responsive promoter is activated thereby ensuring that only leaky expression results in degradation to minimise the impact on the maximum expression.^1^, ^65^

Leaky expression can be reduced post‐transcriptionally through antisense transcription where another promoter downstream of the protein transcribes the non‐coding strand to produce complementary RNA to the mRNA being transcribed from the leaky promoter.^66^ This can be used to generate double stranded RNA which can trigger degradation of the RNA or prevent translation by blocking the ribosome, shown in Figure 8b. The strength of the promoter used to generate the antisense RNA will alter how much of the mRNA has complementary RNA bound, interfering with it being translated. Antisense transcription can also directly interfere with transcription of the mRNA.

Operator sites can be altered to reduce transcription without activation. For activators where the transcription factor is able to bind even without the target, decoy binding sites have been used to direct this binding away from the responsive promoter.^8^ This can reduce leakiness by preventing binding to the responsive promoter to activate expression. Conversely in repressors this will reduce the amount of repressor able to bind to the promoter, shown in Figure 8c. The number of binding sites will alter how much of the transcription factor is titrated away.^67^ This can also affect the LOD because it changes the level of available transcription factor. For repressor based biosensors the operator sites within the promoter can be optimised to improve the promoter response and reduce leaky expression from the promoter at high target concentrations. The position of the operator will alter the strength of the repression generated, so by moving the position of the operator potentially the repression can be strengthened or weakened. Repression is strongest at the core site between −10 and −35 sites, and if there is overlap with one of these sequences this improves repression further,^59^ followed by the proximal site (downstream of the promoter), then the distal site upstream of the core site,^68^ shown in Figure 8di. Adding additional operator sites downstream of the promoter can improve repression if there is still leakiness by acting as a physical block to the polymerase^69^ Figure 8dii. Operator sites have also been altered in eukaryotic cell‐based biosensors to improve the sensitivity.^70^

## 5 Increasing Sensing Selectivity

Many natural transcription factors are promiscuous because this allows cells to respond to more molecules without the requirement for sensing proteins for each molecule.^71^ Non‐specific sensors are a good method for initial screening of groups of molecules.^72^ However the majority of biosensors need to be highly specific to the target to ensure the results are reliable and accurate. Binding to alternative molecules can lead to a false positive if this alternative still activates the responsive promoter. Or if the non‐specific interaction results in inactivation of the protein then this can reduce the response of the sensor leading to inaccurate quantification.

Directed evolution to alter the structure of transcription factors has also been used to improve the selectivity of transcription factors by altering the binding pocket. Improving the binding of a protein to its target has been more successful than altering the specificity to a new target, but it is still a slow process.^73^ Improving the specificity of a transcription factor also usually improves the sensitivity of the biosensors because altering the binding site to bind better tightens the binding of the ligand to the transcription factor.^73^

**Figure 9:**
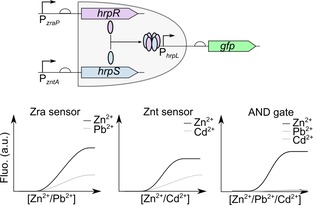
Integrating multiple responsive promoters to increase sensing selectivity: An AND logic gate can be used to generate a highly selective biosensor for zinc. Each zinc responsive promoter responds to another metal ion, promoter P_zraP_ also responds to lead and the promoter P_zntA_ also responds to cadmium. The output promoter P_hrpL_ is activated by a protein complex formed from two different subunits (HrpR and HrpS). By splitting the expression of these two components under the control of different target responsive promoters (P_zraP_ and P_zntA_) this ensures that reporter (GFP) expression only occurs when both promoters are activated. Activation of both promoters will only occur in the presence of zinc and not with either of the non‐specific molecules (lead and cadmium).^28^

Genetic circuits built using logic gates have been used as a method to improve the selectivity. Where multiple parts exist capable of sensing a target but with different promiscuity these can be combined into an AND gate where both sensors need to be activated to generate the response, Figure 9.^28^ Logic gates can also be used to detect multiple targets of interest^28^, ^74^, ^75^ which are all related to a specific condition of interest. This approach is useful in diagnostics where changes in multiple markers is often required.

The identification of more parts which can be used in the development of biosensors has also helped to improve the selectivity that can be achieved as more selective parts can be picked initially for the development of a biosensors.

## 6 Improving Sensor Stability

In order for cell‐based biosensors to meet the requirements for commercialisation the stability of the sensor within the cell is highly important. The output of the sensor construct needs to be maintained despite fluctuations in the environmental conditions to ensure that the results are considered reliable. The sensor constructs themselves also need to be stably maintained within the cells without changes to the construct or loss of function.

When cell‐based biosensors are used to test real samples and testing is carried out in the environment rather than under lab based conditions there will be fluctuations in the environmental conditions during the course of sensing. Ensuring that the sensor is stable and unaffected by these changes is highly important for their acceptance as a reliable alternative to current sensors. Regulatory networks built into the genetic circuits could be used to ensure the response curve is maintained over different conditions. Autoregulation of a protein can be used to reduce variability in the expression of the protein to generate a more consistent concentration of the protein. This could be used when expressing transcription factors to ensure that the changes in the concentration of the transcription factor are limited and so prevent this from impacting the response of a genetic circuit.^76^ More complex regulatory networks have also been engineered. For example feedforward loops could be used to ensure stable expression of the transcription factor due to changes in copy number or expression from the gene to prevent fluctuations in the transcription factor concentration altering the response.^77^

The impact of the genetic circuit constructed for sensing on the cell is important for it to be stably maintained within the cell. Inserting extra genetic information into a host increases metabolic load to the cell diverting nutrients and cellular machinery away from cellular maintenance and growth. If the genetic circuit uses too much of the cell's resources this is a problem for the cell and mutations to reduce or to lose the circuit will occur. Research into quantifying this load and how to reduce the load has been important to develop circuits which minimise their impact on the cell. Notably copy number is an important consideration with the same circuit on medium or low copy number plasmid showing drastically different burdens and output responses.^78^ The development of programmes to model and design genetic circuits that can take into account the issue of burden to design constructs which reduce their impact on the cells can help to reduce instability in the circuits^79^

The long term stability of the entire cell‐based sensor to allow storage between production and use to ensure they can be mass produced is important to commercialise cell‐based biosensors. Currently synthetic biology has not developed tools for extending the shelf life of cell‐based biosensors although other approaches have been studied for storing cell‐based biosensors. Both lyophilisation and encapsulation methods have been studied to generate cell‐based biosensors which can be stored for the required lengths of time needed for commercialisation. There are many examples of lyophilisation to store cell‐based biosensors^2^, ^8^, ^80^, ^81^ however these cells still require specific storage conditions to maintain their activity at either 4 °C^80^ or −20 °C.^81^ Encapsulation or fixation of cells also allows cells to be stored for longer periods of time whilst maintaining activity^82^, ^83^, ^84^ but again requires specific storage conditions.

Bacteria which naturally produce spores can be used to store the sensor for long time periods as these spores can be induced to reanimate when the sensor is required,^85^ with sensor activity retained after 12 months under a range of different conditions including ambient temperature.^86^

Implementation of regulatory networks, such as feedforward loops, can help to ensure that the output from the biosensors at a specific concentration of input remains constant despite fluctuations in environmental conditions keeping the sensor stable under changing conditions. The increased understanding of how additional DNA affects the cell and its health allows the impact to be minimised to prevent selection pressure resulting in loss of sensor function. This shows that the sensor circuits can be designed and constructed with improved stability, although the challenge of improving the shelf life of sensors still needs to be overcome.

## 7 Addressing Sensor Biosecurity

Concerns over the use of genetically modified organisms and their escape into the environment continue to hinder the acceptance of biosensors as an alternative analytical method. There has been a large body of work devoted to the development of control mechanisms within the engineered cells to prevent the escape of the genetic information and the cells into the environment.

Toxin/anti‐toxin systems where the production of a toxin is encoded on the plasmid whilst the anti‐toxin is encoded in the genome of the bacteria being used for the cell‐based biosensor is one method to prevent horizontal gene transfer. This prevents the transfer of the plasmid to other bacteria in the environment because without the ability to produce the anti‐toxin the plasmid will be lethal to the cell.^87^ The use of non‐canonical amino acids is another method to prevent horizontal gene transfer because other bacteria will not contain these alternative amino acids so would not recognise them. The most common approach to using alternative amino acids is to recode bacteria where one of the stop codons is instead used to insert a non‐natural amino acid. The use of a recoded stop codon also helps to prevent horizontal gene transfer because obtaining these genes in an organism which has not been recoded will result in truncated proteins being produced.^88^ This also makes the cell an auxotroph, only able to produce protein when supplemented with the non‐natural amino acid so can be used to prevent cell survival if essential genes are recoded.

The development of methods to prevent the escape of the modified bacteria into the environment has also been studied. One method is to encode kill switches which would result in the death of the cells if they escaped. Multiple different forms of kill switches have been developed to act in different ways. The ‘deadman’ switch uses a toggle switch to control the expression of a toxin so that the toxin was repressed when a small molecule was present, in this case ATc, but loss of this input results in death.^89^ To ensure that killing is effective fail safe modules were also added to directly activate the production of the toxin. Targeted degradation of essential genes on activation of the kill switch was used to improve the killing effect. Although a concern is that selection pressure will result in the loss of the kill switch, work has also developed kill switches with memory elements to ensure that loss of the kill switch results in death of the cells. This uses a toxin/anti‐toxin system where the toxin is not produced when either the memory element or the input signal for the sensor is present but if the memory element is lost or there is no input the result is high expression of the toxin overwhelming the low expression of the anti‐toxin resulting in cell death.^90^

Conditional survival of the cells can also be used to ensure that the cells are unable to survive without intervention by users to maintain the cells in the required conditions such as the ‘passcode’ kill switch.^89^ Temperature control of cell survival has been achieved through controlling the regulation of toxin/anti‐toxin systems so that expression of the toxin is prevented at a desired temperature but if the temperature drops below this, such as if the cells have escaped into the environment, then toxin production rises above what the level of expression of the anti‐toxin can handle and results in cell death.^90^

Combinations of these systems have been used to generate cells which not only prevent horizontal gene transfer of the plasmid to other cells but ensure that the cells themselves are not able to survive in the environment without the addition of nutrients.^87^ In one example the initiators for the origins of replication of the plasmids are also located on the chromosome to prevent replication of the plasmid outside of the engineered host. Essential genes are also deleted to make the cells auxotrophic and toxin/anti‐toxin systems are employed to prevent horizontal gene transfer. Other layered systems have been developed based on auxotrophy through regulating essential genes using small molecules which cannot be supplemented in the environment through cross feeding to prevent escape. Multiple genes were chosen to reduce the chance of escape through mutations. Then biotoxic modules were also engineered into the cell to ensure that the cells are not able to persist in the environment.^91^ The layering of multiple controls reduces the chance of escape mutants even further because several mutations would be required to deactivate all of the controls.

These control mechanisms have been shown to be able to prevent escape of cells into the environment and shown to be stable. This ensures the measures are passed down through generations and for the entire usage of the cell‐based biosensors these control mechanisms remain present. However public opinion towards genetically modified organisms is still highly negative and these biosecurity methods have not been enough to ensure confidence in genetically modified organisms for commercialisation. Other methods of biosecurity need to also be considered such as using a physical barrier to isolate the engineered bacteria from the environment. This could be used in combination with engineered controls to add another layer of biosecurity to help ease fears.

Cell‐free transcription and translation systems allow for genetically encoded biosensors to carry out their sensing function and produce an output outside of the cell. Such systems could be used to develop biosensors for commercialisation because the lack of replicative machinery ensures that the biosensor cannot escape into the environment. Although methods to prevent the genetic information from cell‐free being taken up by cells in the environment will need to be considered. The generation of a genetic firewall would be considered the ultimate biocontainment tool by generating cells which use XNA rather than DNA to ensure that engineered cells are unable to interact with natural cells.^92^

Most importantly to stop biosecurity fears preventing commercialisation of cell‐based biosensors public acceptance of the use of genetically modified organisms is required. Therefore education and outreach to ease fears and remove regulatory barriers will be crucial.

## 8 Summary and Outlook

Many of the early challenges in developing biosensors surrounding the available parts and tuning the response of the constructed biosensor have been the focus of research, with new tools and approaches successfully developed. Tools to program and alter the response have been developed for each part of the genetic circuits used for biosensing. This means that biosensors are now increasingly quick to construct and optimise as the whole process can be optimised for the particular detection requirements of the sensor. The ability to automate the design and optimise design aspects prior to construction has also helped to speed up the process.

The stability of the sensor and ensuring that results between different tests are consistent is an ongoing area of research in cell‐based biosensors. Work into understanding and minimising the impact of sensor circuits on the host cell are important to ensure that the cells maintain biosensor function. There is now increased understanding of burden and its causes which are now taken into consideration. The development of additional regulation to the sensor designs can be used to ensure that fluctuations in environmental conditions for the cell‐based biosensors which would affect expression of the biosensor components do not impact the final result. More work is needed to develop and improve storage methods and cell‐free freeze drying and spore generation currently seem to offer the best new approaches. Future work will need to be directed at achieving storage stability in a commercial viable manner which is competitive in comparison to alternative methods available.

Biosecurity has been a large focus and consideration in synthetic biology research with lots of methods shown to be able to be stably maintained within the cells and effectively kill the cells to ensure there is no escape into the environment. Public acceptance is still an ongoing issue despite significant progress being made to ensure that cells and the genetic constructs contained within them are not able to survive within the environment.

Another important consideration in developing biosensors commercially is how the results are determined. The potential advantages of ease of use and use outside of a lab without specialist equipment were a large factor for the interest in biosensors. The development of smartphones and advances in camera sensitivity has allowed them to be used as portable equipment for detection of reporters found in biosensors. The main challenge to developing methods for detecting the response of biosensors is that portable methods are far less sensitive than lab equipment. Whilst improved data processing and collection methods have helped to improve the quality and reliability of the data collected using smartphones, the requirements of the biosensors in terms of signal‐to‐noise ratio and sensitivity are much higher and require more rigorous optimisation and characterisation.

Recent works into overcoming the ongoing challenges show that significant progress has been made into biosecurity and improving the stability of sensors. Further work is needed to further improve the stability particularly the long‐term shelf life of cell‐based biosensors so that they are able to compete with current commercial sensors and be seen as a viable alternative. The output from the biosensor and how the results from the sensor are determined is another aspect of cell‐based biosensors that still needs to be considered when developing cell‐based biosensors to ensure ease of use which will help increase uptake of cell‐based biosensors as a commercial alternative.

## Conflict of interest

The authors declare no conflict of interest.

## Biographical Information

*Maggie Hicks joined Dr Baojun Wang's lab as a PhD student in October 2018 after receiving her MRes in Systems and Synthetic biology from Imperial College London in 2018 and her BSc in Biochemistry from the University of Bristol in 2017. Her work focusses on the construction of synthetic biology enabled cell‐based biosensors for detecting health related signals for the development of health monitoring devices*.

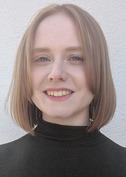

## Biographical Information

*Till Bachmann is Professor of Molecular Diagnostics and Infection, Deputy Head of Infection Medicine, Director of Clinical Microbiology and Infectious Diseases MSc and Biomedical Sciences PhD programmes at the University of Edinburgh and the Zhejiang University – University of Edinburgh Institute in China. Till has a PhD on biosensors from research at University of Stuttgart and University of Tokyo. He is an expert in point‐of‐care detection of infectious diseases and antimicrobial resistance. He is Vice‐Chair of the Scientific Advisory Board of the Joint Programming Initiative on Antimicrobial Resistance, member the UK AMR Diagnostic Collaborative, Panel Member for the Longitude Prize on Antibiotics and founder of AMR DxC*.

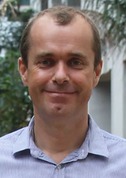

## Biographical Information

*Dr Baojun Wang is Reader in Synthetic Biology and a UK Research and Innovation Future Leaders Fellow at the University of Edinburgh. He leads the Synthetic Biological Circuit Engineering Lab in the School of Biological Sciences and the cross‐disciplinary Edinburgh Centre for Synthetic and Systems Biology. He received a PhD in Bioengineering from Imperial College London (2011) and was a Research Associate at Imperial College before joining Edinburgh University in 2013 as a Group Leader and Chancellor's Fellow in Synthetic Biology. His research interests include building novel customized genetic circuits for sensing, information processing and computing of multiple cellular and environmental signals with applications in diverse areas, for example, biosensing, biocomputing, biomanufacturing and biotherapies*.

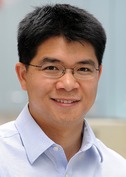

## Supporting information

As a service to our authors and readers, this journal provides supporting information supplied by the authors. Such materials are peer reviewed and may be re‐organized for online delivery, but are not copy‐edited or typeset. Technical support issues arising from supporting information (other than missing files) should be addressed to the authors.

Supplementary
