# Supplementary material for: Synthetic Biology Enables Programmable Cell‐Based Biosensors
Source: Chemphyschem. 2019 Oct 25;21(2):132–44. doi: 10.1002/cphc.201900739 (PMC7004036; doi:10.1002/cphc.201900739)
Supplement: Supplementary file 1 — Supplementary [file CPHC-21-132-s001.pdf]

Author Contributions

B.W. Writing - Original Draft:Equal; Writing - Review & Editing:Lead
M.H. Writing - Original Draft:Equal
T.B. Writing - Review & Editing:Equal
